# Renal resistive index at ICU admission and its change after 24 hours predict acute kidney injury in sepsis

**DOI:** 10.1186/cc13556

**Published:** 2014-03-17

**Authors:** I Gornik, A Godan, V Gašparović

**Affiliations:** 1 University Hospital Centre Zagreb, Croatia

## Introduction

The renal resistive index (RI) measured by Doppler ultrasound reflects the changes in renal microvascular resistance. It was recently shown that the RI measured at ICU admission was associated with development of AKI and with persistent AKI on day 3 after admission. The aim of this study was to investigate whether change of RI after the first 24 ICU hours has an additional predictive value for the development of AKI.

## Methods

This non-interventional study included adult patients with sepsis admitted to a medical ICU. Patients with renal transplant and those in terminal renal failure were excluded. The RI was measured within 2 hours from admission and 24 ± 2 hours after ICU admission. Occurrence of AKI within the first 5 days was classified according to the AKI Network criteria. There was no intervention guide by RI measurements, nor were the results known to the attending physicians.

## Results

There were 52 patients included in the study. At admission, eight patients had AKI stage 2 or 3 and it was persistent in five patients on day 3. Within the first 5 days a total of 17 patients had AKI stage 2 or 3. The RI at admission was associated with APACHE II score, hypotension at admission, baseline serum lactate and history of type II diabetes treated with insulin. Patients who developed AKI stage 2 or 3 had significantly higher RI on admission (0.79 vs. 0.67, *P *= 0.021). A cutoff value of 0.72 best differentiated patients with AKI 2 or 3. Raise of RI by ≥0.05 during the first 24 hours was associated with the development of AKI stage 2 or 3 (*P *= 0.034). Patients with persistent AKI on day 3 had higher RI values on admission, and those who recovered lowered their RIs by >0.05. In multivariate analysis, admission RI and raise of RI by ≥0.05 were independently associated with development of AKI stage 2 or 3 and ICU mortality. Best sensitivity for AKI was achieved when both criteria were used: elevated RI at admission (>0.72) and raise of RI by ≥0.05 at 24 hours post admission.

## Conclusion

The renal RI measured at admission and its dynamics after the first 24 ICU hours are predictive of the development of AKI and ICU mortality. Further studies are needed to confirm these results.

